# Acute Effects of Heel-to-Toe Drop and Speed on Running Biomechanics and Strike Pattern in Male Recreational Runners: Application of Statistical Nonparametric Mapping in Lower Limb Biomechanics

**DOI:** 10.3389/fbioe.2021.821530

**Published:** 2022-01-28

**Authors:** Peimin Yu, Yuhuan He, Yaodong Gu, Yuwei Liu, Rongrong Xuan, Justin Fernandez

**Affiliations:** ^1^ Faculty of Sports Science, Ningbo University, Ningbo, China; ^2^ Research Academy of Grand Health, Ningbo University, Ningbo, China; ^3^ Auckland Bioengineering Institute, The University of Auckland, Auckland, New Zealand; ^4^ Obsterical Department, The Affiliated Hospital of Medical School of Ningbo University, Ningbo, China; ^5^ Department of Engineering Science, The University of Auckland, Auckland, New Zealand

**Keywords:** heel-to-toe drop, strike index, statistical nonparametric mapping, loading rate, footwear

## Abstract

With the increased popularity of running, many studies have been conducted into footwears that are highly related to running performance and running-related injuries. Previous studies investigated different shoe types and running shoes with different heel-to-toe drops (HTDs). However, no research was found in investigating shoes with negative values with HTD. Therefore, the aim of this study was to determine the acute effect of HTD and running speed on lower limb biomechanics and strike pattern in recreational runners. Thirteen male recreational runners wearing shoes with two different HTDs (−8 and 8 mm) performed running at three different speeds (preferred speed [PS], 90% of PS, 110% of PS). Lower extremity kinematics and ground reaction forces were synchronously captured *via* Vicon motion analysis system and AMTI force platform. Strike index (SI), vertical average loading rate (VALR), vertical instantaneous loading rate (VILR), excursion, eversion duration, joint angles, and range of motion (ROM) of metatarsophalangeal (MTP), ankle, knee, and hip joints were calculated. Joint angles during the entire stance phase were analyzed applying the statistical nonparametric mapping (SnPM) method. SI and VILR in shoes with −8 mm HTD significantly increased by 18.99% and 31.836 BW/s compared to those with 8 mm HTD (SI: *p* = 0.002; VILR: *p* < 0.001). Significant alterations of ROM occurred in the MTP, ankle, and knee joints (*p* < 0.05), and HTD factor primarily accounted for these changes. Joint angles (MTP, knee, and hip) during the entire stance phase altered due to HTD and speed factors. Running speed primarily influenced the kinematics parameters of knee and hip joints, increasing knee angles in the frontal plane and hip angle in the horizontal plane at PS (*p* > 0.05). Compared to shoes with 8 mm HTD, shoes with −8 mm HTD may be useful to storage and return energy because of the increased ROM of MTP in the sagittal plane. Besides, forefoot strike gait retraining was recommended before transition from normal running shoes to running shoes with −8 mm HTD.

## 1 Introduction

Running, as one of the convenient, low-cost, and beneficial sports, is widely welcomed by people of all age groups among the world (Haskell et al., 2007; Fields et al., 2010). Accompanied with the popularity of running, more people were involved in running, and the risk of skeletal muscle injuries is also increased. Almost a half of runners would have a history of running-related injuries each year (Fields et al., 2010). Among all running-related injuries, the most common injury types are patellofemoral pain syndrome, iliotibial band friction syndrome, plantar fasciitis, meniscal injuries of the knee, and patellar tendinopathy (Taunton et al., 2002). To reduce the risk of running-related injuries, different types of running shoes were designed, which are minimalist, maximalist, and traditional running shoes (Chan et al., 2018; Mo et al., 2020; Mo et al., 2021). Both minimalist and maximalist running shoes had a shared feature of low heel-to-toe drop (HTD) compared to traditional running shoes (Malisoux et al., 2016; Mo et al., 2021). Over a period, maximalist running shoes were supposed to reduce the impact loading *via* the highly cushioned midsole (O’Leary et al., 2008). However, it remained controversial whether enhancing the shock absorption performance of running shoes could reduce the running-related injuries (Yan et al., 2013).

The foot strike patterns could be divided into forefoot strike (FFS), midfoot strike (MFS), and rearfoot strike (RFS), which would result in difference of the vertical loading rate, and thus influence the running-related injury risk (Daoud et al., 2012). Habitually barefoot runners usually adopted the FFS pattern, while habitually shod runners maintained the RFS pattern during running (Lieberman et al., 2010; de Almeida et al., 2015). Minimalist running shoes were produced to imitate the barefoot running pattern, since the idea that barefoot running might be beneficial to reduce the risk of running-related injuries was put forward (Lieberman et al., 2010). Minimalist running shoes took characteristic as low midsole stack height, high flexibility, and low HTD and weight (Esculier et al., 2015). In some studies, it is found that wearing minimalist running shoes could imitate the running biomechanics of barefoot running (Squadrone and Gallozzi 2009; Sinclair et al., 2013b; Paquette et al., 2013), or that it could approximate the barefoot running pattern after certain practice and adaptation (Rixe et al., 2012), while others held the opinion that the biomechanical characteristics of the lower limb were similar between minimalist running shoes and traditional running shoes (Bonacci et al., 2013; Frank et al., 2013; Bergstra et al., 2015). The differences of these studies might be caused by the usage of various types of minimalist running shoes, since HTD of minimalist running shoes varied from 0 to 8 mm (Horvais and Samozino 2013). HTD, as a main feature of shoe design, was associated with alterations of lower limb biomechanics. Reduced HTD led to a transition of the foot strike pattern from RFS to FFS or MFS and changes of ankle and knee joints kinematics and kinetics (Horvais and Samozino 2013; Lee et al., 2013; Richert et al., 2019). As for the injury risk, although the injury risk among all runners was not changed, lower HTD shoes were associated with the lower injury risk among occasional runners, but the higher injury risk among regular runners (Malisoux et al., 2016).

Running speed is also one of the factors that influence the foot strike pattern and lower limb biomechanics of runners (Mo et al., 2021). Cheung et al. found that the proportion of FFS and MFS patterns increased with the increasing of the running speed (Cheung et al., 2017), whereas in the study of Fredericks et al., the foot strike pattern was not obviously influenced by the running speed (Fredericks et al., 2015). As for the kinematics of the lower limb, the influence of running speed mainly focused on the knee joint, resulting in the increase of the flexion angle at the initial contact (Bishop et al., 2006). Moreover, with the running speed increasing, the peak values of hip flexion and extension increased. With regard to the ankle joint, the dorsiflexion angle increased at the initial contact, and the peak plantar flexion angle increased during the early swing phase (Orendurff et al., 2018). With regard to the kinetics of the lower limb, the vertical instantaneous loading rate (VILR) increased when running speed increased (Breine et al., 2019).

To date, there is a lack of information about the effects of negative HTD on foot strike pattern and lower limb biomechanics in recreational runners. Furthermore, the influence of running speed was controversial because of different values of speeds. More information was required to illustrate the relationship among running speed, HTD, and lower limb biomechanics. Therefore, the aim of the current study was to investigate the effects of HTD and running speed on lower limb biomechanics and strike pattern in recreational runners. It is hypothesized that 1) running shoes with the negative value of HTD would alter the lower limb biomechanics and the foot strike pattern of runners, the proportion of FFS and MFS might increase; 2) increasing running speed would mainly affect the loading rates and kinematics of the lower limb rather than the foot strike pattern.

## 2 Methods

### 2.1 Participants

Prior to the study, the sample size of the current study was calculated *via* G*Power 3.1.9.7 (effect size = 0.4, *α* value = 0.05, power value = 0.8) (Kang 2021). A total of 13 male recreational runners (age 23.67 ± 1.21 years, height 169 ± 5.59 cm, weight 60.83 ± 5.87 kg, body mass index 21.27 ± 1.32 kg/m^2^, shoe size 41 EU) were involved in this study. The definition of recreational runners was based on the previous study, which required runners ran less than 3 times per week and less than 10 km per trail (Yu et al., 2021). All participants were rearfoot strikers with the right limb as the dominant limb, defined as the leg used to kick a ball. Individuals with any foot deformity or lower limb injuries in the previous 6 months were excluded from participation. Besides, all participants had no previous running experience in the running shoes with negative value of HTD. To avoid adjusting the foot strike pattern subjectively, participants were not informed of the study aim. Before the experiment, all individuals were given the inform consent, and the ethical approval was granted by the Ningbo University Ethics Committee.

### 2.2 Shoe Conditions

**FIGURE 1:**
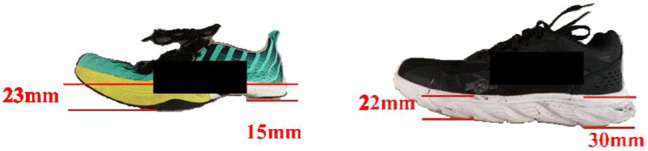
Illustration of shoe types (left side: running shoes with the −8 mm HTD; right side: running shoes with the 8 mm HTD).

All participants conducted this study in shoes with a positive and a negative value of HTD, respectively (Figure 1). These two types of shoes were produced by the same footwear company. The shoe size of these two shoes was 41 EU, midsole material was ethylene-vinyl acetate (EVA), and the outsole material was rubber. Taking the traditional running shoes was the control shoes with the HTD 8 mm, and the height of the sole at the heel and forefoot region was 30 and 22 mm (D8). HTD of the experimental running shoes was −8 mm, and the height of the sole at the heel and forefoot region was 15 and 23 mm (D-8).

### 2.3 Testing Procedure

All participants were given 5 min to warm up and be familiar with the study environment. The preferred speed (PS) of each subject was recorded *via* Brower timing light (Brower Timing System, Draper, UT, United States). Based on the PS, 90% of the PS was denoted as comparatively slower speed (90% PS), and 110% of the PS was denoted as comparatively quicker speed (110% PS) (Van Oeveren et al., 2017). Participants were required to wear these two types of running shoes to conduct running tasks with three different running speeds (90% PS, 100% PS, 110% PS) in the laboratory. Each subject conducted three successful trials of running test under the different running speeds and shoes. Average values of these three trails were used to minimize the inter-trail error. To avoid the influence of fatigue, each trial was required 30 s interval. The sequence of the shoe types and running speeds was random.

**FIGURE 2:**
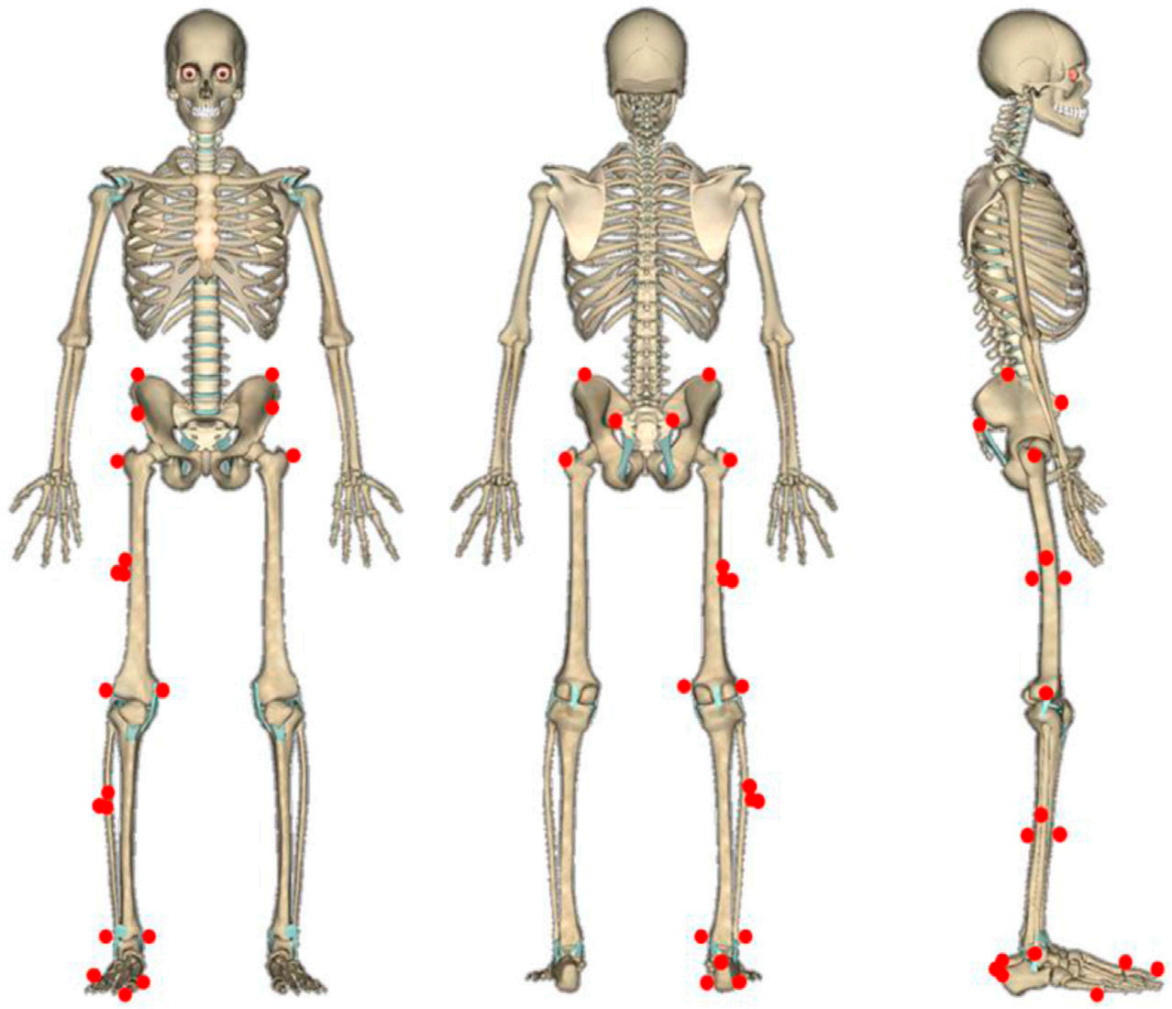
Illustration of reflective markers on subjects. From left to right: front view, rear view, and right side view.

Lower extremity kinematics was captured *via* the 8-camera motion analysis system (Oxford Metrics Ltd., Oxford, United Kingdom) with a frequency of 200 Hz. All subjects were required to wear tight-fitting pants, and 24 reflective markers with a diameter of 12 mm were used to define the motion axis and the center of the lower limb joint (Zhu et al., 2020). The reflective markers were attached unilaterally (right limb) except the pelvis segment, using double side tape to attach on the following anatomical landmarks (Figure 2): left/right iliac crest, left/right anterior superior iliac spine, left/right posterior–superior iliac spine, left/right greater trochanter (Cen et al., 2021), three thigh tracking targets, femoral lateral/medial epicondyle, three shank tracking targets, lateral/medial malleolus, three heel tracking targets, first and fifth metatarsal heads, and toe. Based on the previous studies, the metatarsophalangeal (MTP) joint angle referred to the angle between the forefoot and the rearfoot coordinate system, and the rotation axis referred to the center of the first and fifth metatarsal heads and the conjunction between the reflective markers of the first and fifth distal metatarsals (Zhu et al., 2020; Cen et al., 2021). The AMTI force platform (Advanced Mechanical Technology, Inc., Watertown, MA, United States) was used to synchronously capture the ground reaction forces (GRFs) with a sample frequency of 1,000 Hz. Prior to the data collection, the force platform was zero-leveled, and the stance phase was defined through the vertical GRF with the threshold set to 20 N.

### 2.4 Data Processing

Experimental data of each trail during the stance phase were processed and analyzed in the current study *via* Visual3D software (version 3.26; C-motion Inc., Germantown, MD, United States). The trajectory of reflective markers was filtered by a low-pass filter with a cut-off frequency of 10 Hz (Yu et al., 2020). The joint angles were calculated *via* an inverse kinematics algorithm in Visual3D and normalized to 100 frames. Stance phase was defined as the period when the value of vertical GRF surpassed 20 N. Kinematic data of interest included the joint angles (MTP, ankle, knee, hip) in the three planes (sagittal, frontal, transverse) during the stance phase. Range of motion (ROM) was defined as the difference between the maximum and minimum joint angles during the stance phase. Besides, excursion of the ankle joint was also included, which was defined as the difference between the angle at the initial contact and the maximum joint angle (Hannigan and Pollard 2020). The eversion duration was referred to the percentage of the stance phase in which the foot was in an everted position. Kinetic variables included the vertical average loading rate (VALR) and VILR that were calculated based on the previous studies (Milner et al., 2006; Law et al., 2019). When the impact peak was undetectable or absent, the vertical GRF at 13% stance phase was defined as the impact peak (Blackmore et al., 2016). Strike index (SI) was used to classify the foot strike pattern, calculated based on the following equation (Cavanagh and Lafortune 1980).

$$\mathrm{SI}=\frac{D_{\mathrm{COP-heel}}}{\mathrm{foot}\;\mathrm{length}}\times 100\%$$

Where the *D*_cop-heel_ refers to the distance from the location of the center of pressure at the initial contact to the heel along the foot long axis. The SI of 0%–33% presents the RFS pattern, between 34% and 67% is classified as the MFS pattern, and 68%–100% is defined as the FFS pattern (Cavanagh and Lafortune 1980).

### 2.5 Statistical Analysis

Normal distribution was checked for all discrete values. Mean and standard deviation (SD) were applied to describe the discrete values of ROM, excursion, eversion duration, SI, AVLR, and IVLR. These parameters were evaluated by the two-factor repeated measures ANOVA followed by Bonferroni post hoc test for multiple comparisons. Analysis was conducted in SPSS (version 25; SPSS, Chicago, IL, United States). Due to the one-dimensional time-varying characteristic of the joint angle, the two-factor repeated measures ANOVA in open-source factorial statistical nonparametric mapping (SnPM) was employed, which is based on label permutation tests (Nichols and Holmes 2001; Trama et al., 2021). Bonferroni correction was used to adjust the post hoc tests alpha risk (Trama et al., 2021). Factorial SnPM was conducted in MATLAB R2018a (The MathWorks, MA, USA). The significance level was set as 0.05.

## 3 Results

Results that had no significant differences between two factors were presented in the Supplementary Material.

### 3.1 Strike Index

As is shown in Table 1, there was no significant interaction between running speed and HTD on the SI (*p* = 0.269). There was no significant main effect of running speed on foot SI, while the main effect of HTD on foot SI was significant (F (1,12) = 15.040, *p* = 0.002). The foot strike pattern in D-8 shifted anteriorly to 18.99% compared to that in D8 (95% CI: 8.31%–29.61%, *p* = 0.002).

**TABLE 1:** Strike pattern, ground reaction force, and kinematic data for the two shoes under different running speeds.

		D-8			D8			–		
		90% PS	100% PS	110% PS	90% PS	100% PS	110% PS	Interaction	HTD	Speed
Strike index (%)		42.43 (24.83)	42.28 (26.04)	40.47 (25.02)	16.10 (7.80)	22.17 (18.56)	30.05 (26.93)	0.269	0.002^a^	0.198
Ground reaction forces										
VILR (BW/s)		102.20 (32.22)	116.04 (44.95)	119.98 (35.35)	83.35 (23.20)	81.36 (23.90)	78.00 (21.92)	0.242	<0.001^a^	0.466
ROM (°)										
MTP	Sagittal	28.14 (10.86)	5.47 (2.55)	27.54 (13.00)	34.09 (13.81)	30.67 (14.53)	30.33 (13.61)	0.001^b^	–	–
	Frontal	14.68 (7.88)	16.68 (7.96)	13.50 (5.67)	9.86 (5.68)	10.92 (7.09)	10.81 (6.99)	0.001^b^	–	–
Ankle	Frontal	16.87 (7.87)	17.86 (5.62)	15.11 (6.67)	12.40 (4.72)	13.09 (5.77)	13.47 (6.26)	0.069	0.001^a^	0.078
Knee	Horizontal	9.77 (5.08)	9.70 (6.19)	9.14 (5.46)	12.25 (6.72)	11.19 (6.55)	9.72 (6.06)	0.003^b^	–	–
	Sagittal	22.88 (3.77)	20.93 (4.75)	19.75 (4.38)	23.50 (2.75)	23.89 (2.90)	22.17 (4.14)	0.285	0.007^a^	0.027^c^
	Horizontal	10.22 (2.78)	10.88 (2.72)	9.52 (2.48)	11.04 (2.29)	11.57 (2.83)	12.03 (3.30)	0.456	0.018^a^	0.018^c^
Excursion (°)		16.03 (9.93)	12.76 (8.89)	15.60 (6.82)	9.47 (3.17)	12.17 (5.87)	13.62 (6.46)	0.094	0.106	0.026^c^

Notes: PS, preferred speed; HTD, heel-to-toe drop; D-8, shoes with the −8 mm HTD; D8, shoes with the 8 mm HTD; VILR, vertical instantaneous loading rate; ROM, range of motion; MTP, metatarsophalangeal joint.

a Indicates the significant main effect of heel-to-toe drop.

b Indicates a statistically significant interaction.

c Indicates the significant main effect of running speed.

### 3.2 Ground Reaction Forces

As for the VILR (Table1), there was no significant interaction between running speed and HTD (*p* = 0.242). There was no significant main effect of running speed on VILR (F (2,24) = 0.674, *p* = 0.519), while the main effect of HTD on the VILR was significant (F (1,12) = 33.847, *p* < 0.001). The VILR in D-8 significantly increased by 31.836 BW/s compared to that in D8 (*p* < 0.001).

### 3.3 Discrete Kinematic Data

All discrete kinematic data with significant differences are presented in Table 1. For the ROM of MTP in the sagittal plane, there was a significant interaction between running speed and HTD (*p* = 0.001). At the 90% and 100% of PS, the ROMs in D-8 significantly reduced by 5.910° and 25.206° compared to those in D8 (F (1,12) = 6.202, *p* = 0.028; F (1,12) = 30.485, *p* < 0.001). As for the frontal plane, there was also a significant interaction between running speed and HTD (*p* = 0.001). At each group of running speed, the ROMs in D-8 significantly increased (90% PS: F (1,12) = 11.821, *p* = 0.005; 100% PS: F (1,12) = 46.663, *p* < 0.001; 110% PS: F (1,12) = 5.873, *p* = 0.032). In D-8, there was a significant simple main effect of running speed (F (2,24) = 3.475, *p* = 0.047), while the post hoc tests revealed no significant differences between groups (*p* > 0.05). In D8, there was no significant simple main effect of running speed (F (2,24) = 0.598, *p* = 0.468).

For the ROM of the ankle joint in the frontal plane, there was no significant interaction between running speed and HTD (*p* = 0.069). There was no significant main effect of running speed on the ROM (F (2,24) = 2.840, *p* = 0.078), while the main effect of HTD was significant (F (1,12) = 35.097, *p* < 0.001). The ROM in D-8 was significantly greater than that in D8 (*p* < 0.001). As for the horizontal plane, there was a significant interaction between two factors (*p* = 0.003). The significant differences only existed in groups of 90% and 100% of PS, and the ROMs in D-8 significantly reduced by 2.480° and 1.485° compared to those in D8 (F (1,12) = 8.717, *p* = 0.012; F (1,12) = 6.585, *p* = 0.025). As for the ankle excursion, no HTD * speed interaction effect or main effect of HTD was observed (*p* = 0.094, *p* = 0.106). The main effect of running speed was significant (F (2,24) = 4.278, *p* = 0.006). The ankle excursion at 100% of PS significantly reduced by 2.142° compared to that at 110% of PS (*p* = 0.016).

For the ROM of the knee joint in the sagittal plane, there was no significant interaction between running speed and HTD (*p* = 0.285). There was a significant main effect of HTD on the ROM (F (1,12) = 10.454, *p* = 0.007), and the ROM in D-8 significantly reduced by 1.999° (*p* = 0.007). Besides, there was a significant main effect of running speed on the ROM (F (2,24) = 4.665, *p* = 0.027). The ROM at 90% of PS was significantly greater than that at 110% of PS (*p* = 0.032). As for the horizontal plane, no HTD * speed interaction effect was observed (*p* = 0.456). The significant difference only existed in the HTD group; ROM in D-8 significantly reduced by 0.884° (*p* = 0.018).

### 3.4 Joint Angles During the Entire Stance Phase

**FIGURE 3:**
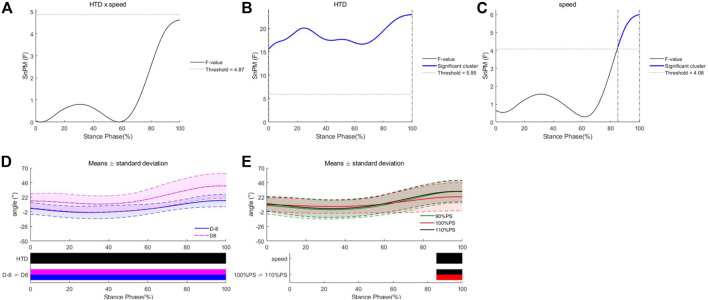
Differences of MTP angles in the sagittal plane under different HTDs and running speeds. Interaction **(A)**, main effects of HTD **(B)** and speed **(C)**, post hoc test for HTD **(D)**, and post hoc test for speeds **(E)**.

**FIGURE 4:**
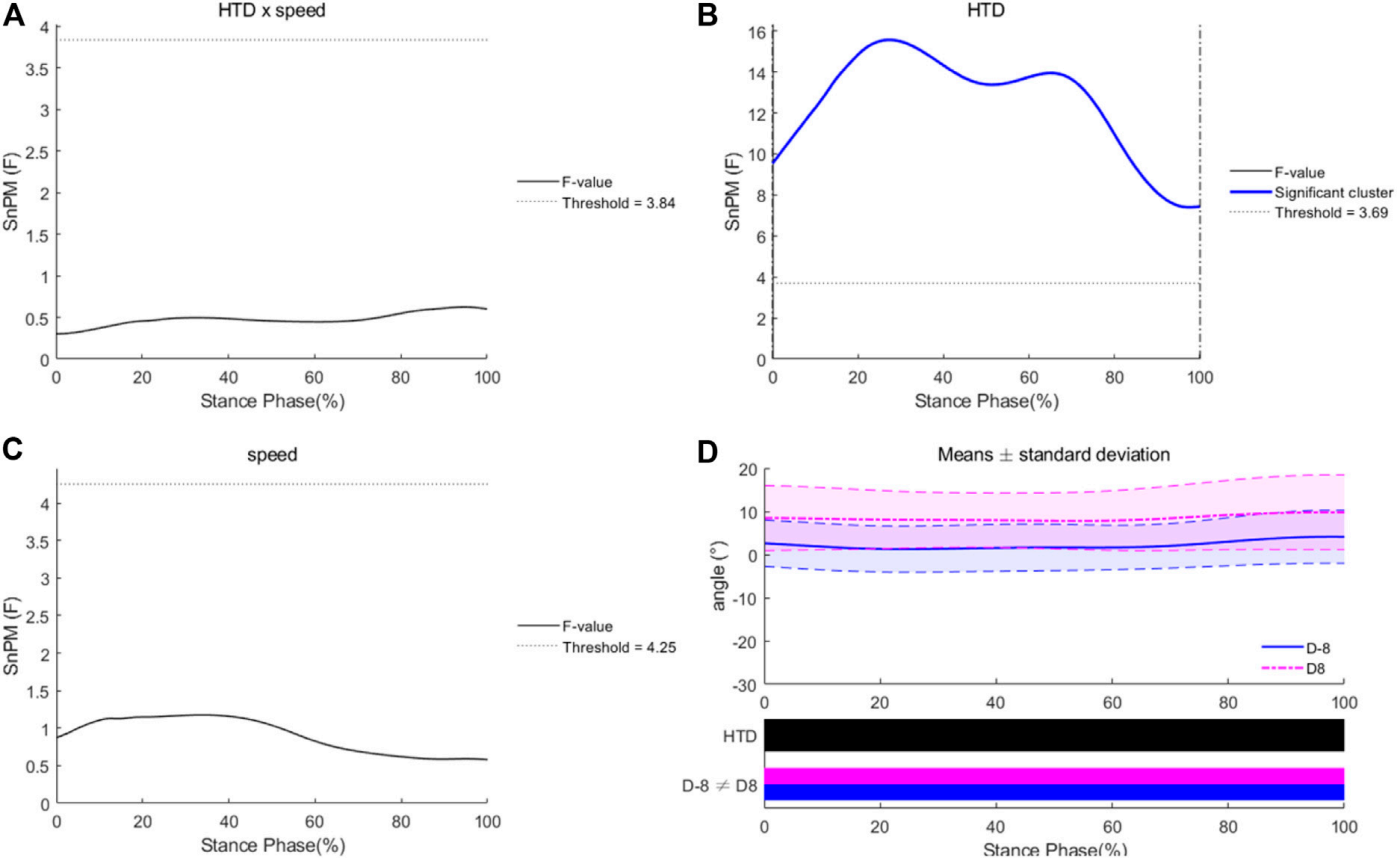
Differences of MTP angles in the horizontal plane under different HTDs and running speeds. Interaction **(A)**, main effects of HTD **(B)** and speed **(C)**, and post hoc test for HTD **(D)**.

No HTD * speed interaction effects were observed for MTP angles in the sagittal plane during the entire stance phase (Figure 3). There was an “HTD” effect, with an F-value above the significant threshold of 5.95 during the entire stance phase. There were significant main effects of running speed for MTP angles in the sagittal plane during 86%–100% of the stance phase, with the F-value above the threshold of 4.08. During that phase, MTP angles at 110% of PS were significantly greater than those at 100% of PS. As for the horizontal plane, no HTD * speed interaction effects or main effects of speed were observed for MTP angles during the entire stance phase (Figure 4). There was an “HTD” effect, with an F-value above the significant threshold of 3.69 during the entire stance phase.

**FIGURE 5:**
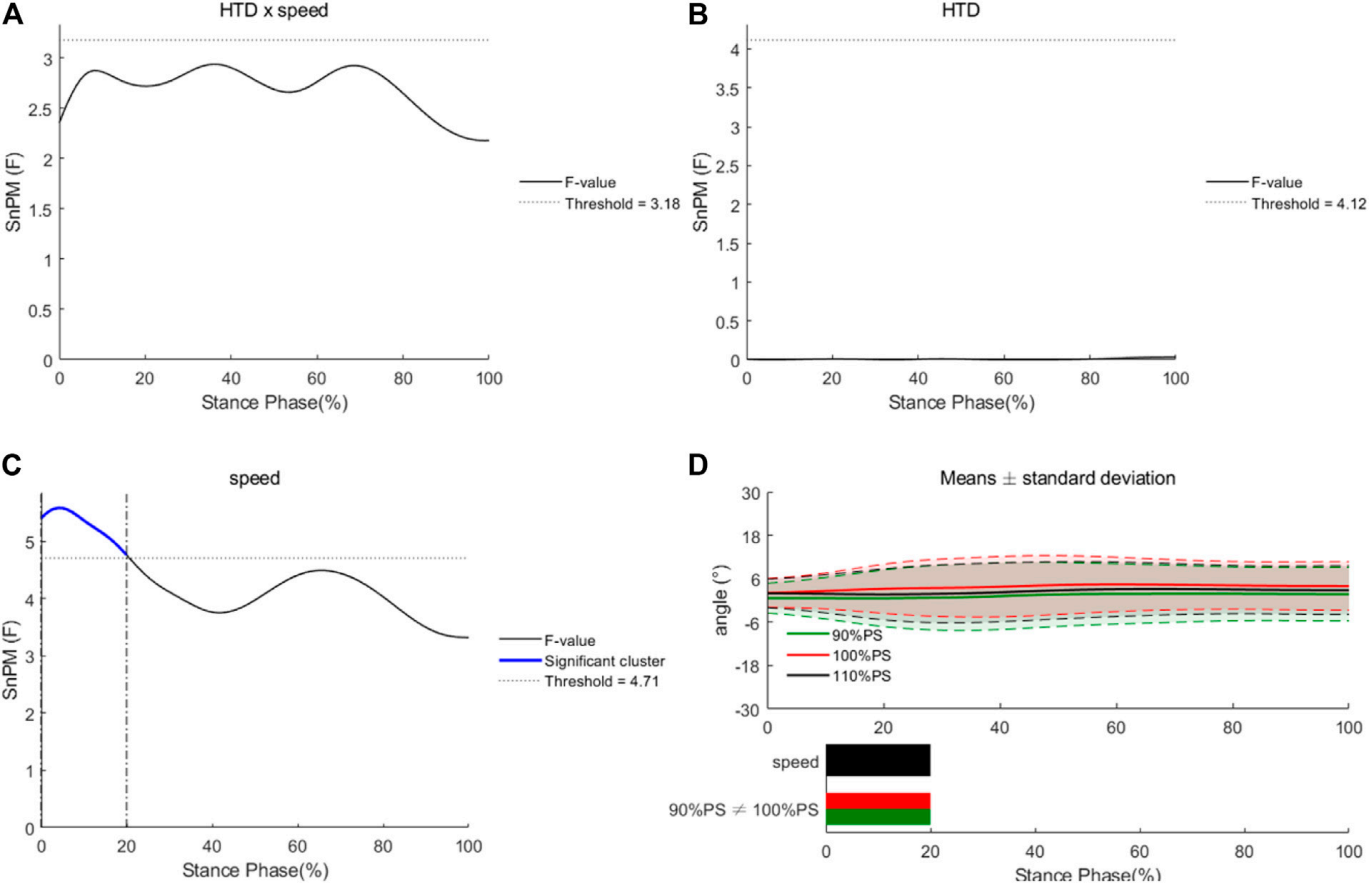
Differences of knee angles in the frontal plane under different HTDs and running speeds. Interaction **(A)**, main effects of HTD **(B)** and speed **(C)**, and post hoc tests for speed **(D)**.

No HTD * speed interaction effects or main effects of HTD were observed for knee angles in the frontal plane during the entire stance phase (Figure 5). There were significant main effects of running speed for knee angles in the frontal plane during 0%–21% of the stance phase. Knee angles at 100% of PS were significantly greater than those at 90% of PS.

**FIGURE 6:**
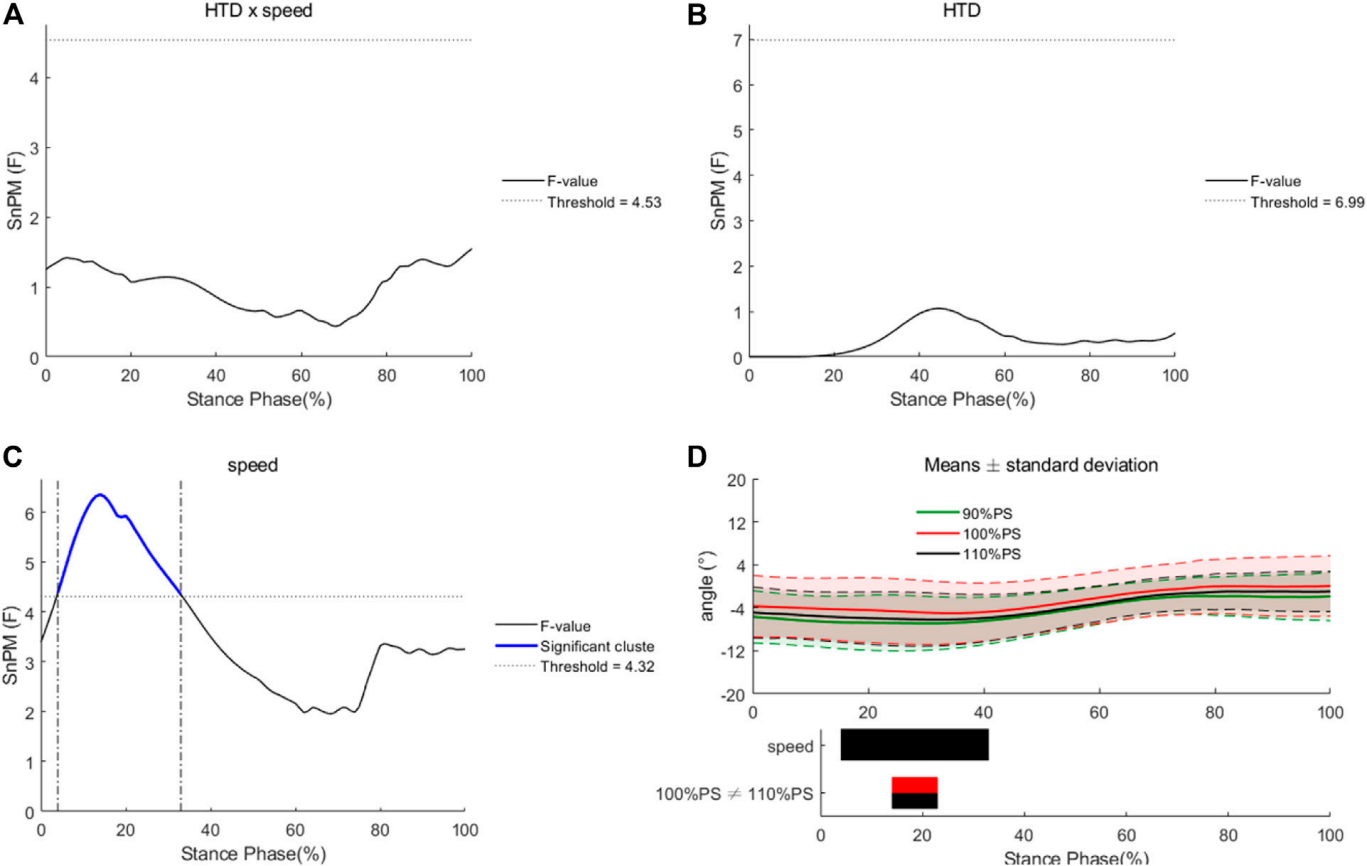
Differences of hip angles in the horizontal plane under different HTDs and running speeds. Interaction **(A)**, main effects of HTD **(B)** and speed **(C)**, and post hoc tests for speed **(D)**.

No HTD * speed interaction effects or main effects of HTD were observed for hip angles in the horizontal plane during the entire stance phase (Figure 6). There were significant main effects of running speed for hip angles in the horizontal plane during 15%–24% of the stance phase. Hip angles at 100% of PS were significantly greater than those at 110% of PS.

## 4 Discussion

The primary aim of the current study was to explore the effects of HTD (−8 and 8 mm) and running speed (90% of PS, 100% of PS, 110% of PS) on lower limb biomechanics and strike patterns in recreational runners. Differences in SI were observed between two different HTDs, and no differences were seen in different running speeds, which did support our hypothesis. Both VALR and VILR were not influenced by running speed, but VILR in D-8 reduced compared to D8, which did not support our initial hypothesis. Running speed or HTD mainly affected the ROM of MTP, ankle, and knee joints. Besides, during the entire stance phase, angles of ankle joint did not change significantly. These findings partially supported our hypothesis.

The influence of running speed on the strike pattern was controversial. While some studies found that the proportion of FFS and MFS patterns increased with the increase of running speed (Breine et al., 2014; Cheung et al., 2017; Jiang 2020), other studies found that it did not change (Fredericks et al., 2015; Lai et al., 2020). The disagreement between these studies might be attributed to different ranges of running speeds, where relatively slow running speed might not influence the strike pattern of runners. The running speeds of the current study were set at 90%, 100%, and 110% of PS. Besides, PS of individuals was relatively slow because this study was conducted in the laboratory environment. Strike pattern, therefore, did not alter with the running speed in the current study. As for the HTD that is a critical feature of the shoe design, it was related with the foot strike pattern (Fredericks et al., 2015; Dempster et al., 2021). To a certain extent, a lower HTD might result in the greater proportion of FFS and MFS patterns according to the previous studies (Hollander et al., 2015; Cheung et al., 2017). Previous studies mainly focused on the comparisons between traditional, minimalist, and maximalist running shoes, all of which have positive values of HTD. In this study, experimental running shoes increased the thickness of forefoot midsole and reduced the thickness of heel midsole, resulting in the −8 mm HTD. Accordingly, the proportion of FFS and MFS patterns increased. Although not all runners used the FFS/MFS pattern, the foot strike pattern shifted anteriorly compared to traditional running shoes.

No significant effects of running speed and HTD were found in VALR. VILR, however, increased in shoes with −8 mm HTD compared to that in 8 mm HTD. GRFs and loading rates (including VALR and VILR) have been applied to investigate the mechanisms of running-induced injuries (Milner et al., 2006; Phan et al., 2017; Breine et al., 2019; Malisoux et al., 2021). Although habitually barefoot runners who commonly adopted the FFS pattern had lower impact peak of GRF and loading rates than habitually shod runners (Lieberman et al., 2010), acute transition to the FFS or MFS pattern in habitually shod runners increased impact peaks and loading rates (De Wit et al., 2000; Willson et al., 2014; Willy and Davis 2014; Hannigan and Pollard 2020). Consistent with the previous studies, VILR in shoes with −8 mm HTD was significantly greater, and even the overall strike pattern shifted anteriorly. The characteristics of participants and shoe design could account for this phenomenon. All participants included in this study were accustomed to the RFS pattern when running. And experimental shoes with −8 mm HTD encouraged an FFS pattern because of the thickness of forefoot midsole. Although the proportion of a non-RFS pattern increased, runners adopted an RFS pattern in some trails of the experiment. To reduce the potential risk of running-induced injuries, FFS gait retraining would be of great importance (Futrell et al., 2020).

The MTP joint, as an essential contributor to lower limb energetics, has also received widespread attention in recent years (Stefanyshyn and Nigg 2000; Roy and Stefanyshyn 2006; Smith et al., 2014). Previous studies have found that MTP joint was a large dissipater of energy, absorbing energy and generating no or very little energy before take-off (Roy and Stefanyshyn 2006; Smith et al., 2014). This could be attributed to dorsiflexion of MTP at the majority phase of the stance and plantar flexion at the end of the stance (Stefanyshyn and Nigg 2000; Willwacher et al., 2013). In this study, MTP angles in the sagittal plane reduced significantly in shoes with −8 mm HTD. The increased thickness of forefoot midsole and decreased thickness of heel midsole promoted runners to perform plantar flexion movement at the stance phase, which was supposed to transfer energy appropriately. When running at 90% and 100% of PS, the ROM of MTP in the sagittal plane in shoes with −8 mm HTD reduced significantly. But the increased ROM of MTP might be beneficial to storage and return energy (Willwacher et al., 2013). More evidence was still required to identify the effects of HTD on energy absorption or production of MTP.

During the entire stance phase, there were no differences of ankle angles between different HTDs or speeds. Consistent with the previous studies, running speed did not significantly influence the ankle angles, which supported the idea that power did not mainly be generated from the ankle joint (Pink et al., 1994; Lohman et al., 2011). In the frontal plane, ROM of ankle joint in shoes with −8 mm HTD was greater than that with 8 mm HTD. That could surmise that shoes with −8 mm HTD produced excessive ankle motion in the frontal plane, which might indicate increased instability of shoes with −8 mm HTD. When running at 90% and 100% of PS, ROM of ankle joint in the horizontal plane decreased when wearing shoes with −8 mm HTD. Besides, dorsiflexion excursion increased at 110% of PS compared to that at 100% of PS. No differences were seen in eversion duration, which has been associated with injury risk of Achilles tendinopathy and medial tibia stress syndrome (Becker et al., 2017).

In the sagittal plane, ROM of knee joint in shoes with −8 mm HTD reduced compared to that with 8 mm HTD. Additionally, the internal–external ROM of knee joint reduced in shoes with −8 mm HTD. During the stance phase, reduced knee ROM was associated with better running economy that was strongly related to running performance (Sinclair et al., 2013a; Moore 2016). Additionally, flexion–extension ROM of knee joint at 110% of PS was greater than that at 90% of PS. In the frontal plane, knee angles at 100% of PS were greater than those at 90% of PS from the first contact to 21% of the stance phase. During that period, runners went from initial contact to loading response. Hip angles in the horizontal plane at 100% of PS were significantly greater than those at 110% of PS during 15%–24% of the stance phase (from heel strike to mid stance). Consistent with the previous studies, knee and hip joints changed with difference running speeds (Pink et al., 1994; Lohman et al., 2011). It could be assumed that knee and hip joints are the main source of power in running rather than ankle joint.

Some limitations of the current study should be noticed. Firstly, although SnPM provided a good approach to compute ANOVA and post hoc tests, the post hoc tests with Bonferroni correction were only approximate and conservative. Furthermore, only immediate effects of HTD and running speed on lower limb biomechanics and strike pattern were compared in this study. The long-term effects of these two factors remained uncertain, which should be considered in the future studies. Finally, participants of this study were all rearfoot strikers, so conclusions might not be appropriate to apply to naturally forefoot or midfoot strikers.

## 5 Conclusion

This study assessed the immediate effects of both HTD and speed on running biomechanics and strike pattern in recreational runners. Foot strike pattern shifted anteriorly when wearing shoes with −8 mm HTD. In addition, VILR in shoes with −8 mm HTD increased, which would increase the risk of injury risks. The influence of HTD on the kinematic parameters mainly happened on the MTP joint, especially resulting in greater joint angles in the sagittal plane. These alterations make storage and return energy effectively compared to shoes with 8 mm HTD. Running speed primarily influenced knee and hip joints that may be the main source of power in running. The results of this study indicated that shoes with −8 mm HTD may have better ability to storage and return energy that was related to running performance. But according to higher VILR in shoes with −8 mm HTD, FFS gait retraining was suggested before transition from normal running shoes to running shoes with −8 mm HTD.

## Data Availability

The original contributions presented in the study are included in the article/supplementary material. Further inquiries can be directed to the corresponding authors.
